# Origins of the current outbreak of multidrug-resistant malaria in southeast Asia: a retrospective genetic study

**DOI:** 10.1016/S1473-3099(18)30068-9

**Published:** 2018-03

**Authors:** Roberto Amato, Richard D Pearson, Jacob Almagro-Garcia, Chanaki Amaratunga, Pharath Lim, Seila Suon, Sokunthea Sreng, Eleanor Drury, Jim Stalker, Olivo Miotto, Rick M Fairhurst, Dominic P Kwiatkowski

**Affiliations:** a Wellcome Sanger Institute, Hinxton, UK; b MRC Centre for Genomics and Global Health, Big Data Institute, Oxford University, Oxford, UK; c National Institute of Allergy and Infectious Diseases, National Institutes of Health, Rockville, MD, USA; d National Center for Parasitology, Entomology, and Malaria Control, Phnom Penh, Cambodia; e Mahidol-Oxford Tropical Medicine Research Unit, Mahidol University, Bangkok, Thailand

## Abstract

**Background:**

Antimalarial resistance is rapidly spreading across parts of southeast Asia where dihydroartemisinin–piperaquine is used as first-line treatment for *Plasmodium falciparum* malaria. The first published reports about resistance to antimalarial drugs came from western Cambodia in 2013. Here, we analyse genetic changes in the *P falciparum* population of western Cambodia in the 6 years before those reports.

**Methods:**

We analysed genome sequence data on 1492 *P falciparum* samples from 11 locations across southeast Asia, including 464 samples collected in western Cambodia between 2007 and 2013. Different epidemiological origins of resistance were identified by haplotypic analysis of the *kelch13* artemisinin resistance locus and the *plasmepsin 2–3* piperaquine resistance locus.

**Findings:**

We identified more than 30 independent origins of artemisinin resistance, of which the KEL1 lineage accounted for 140 (91%) of 154 parasites resistant to dihydroartemisinin–piperaquine. In 2008, KEL1 combined with PLA1, the major lineage associated with piperaquine resistance. By 2013, the KEL1/PLA1 co-lineage had reached a frequency of 63% (24/38) in western Cambodia and had spread to northern Cambodia.

**Interpretation:**

The KEL1/PLA1 co-lineage emerged in the same year that dihydroartemisinin–piperaquine became the first-line antimalarial drug in western Cambodia and spread rapidly thereafter, displacing other artemisinin-resistant parasite lineages. These findings have important implications for management of the global health risk associated with the current outbreak of multidrug-resistant malaria in southeast Asia.

**Funding:**

Wellcome Trust, Bill & Melinda Gates Foundation, Medical Research Council, UK Department for International Development, and the Intramural Research Program of the National Institute of Allergy and Infectious Diseases.

## Introduction

The first-line treatment for *Plasmodium falciparum* malaria is artemisinin combination therapy.^1^ Artemisinin and its derivatives are potent and fast-acting antimalarial drugs, but they are also short acting.^2^ The rationale behind artemisinin combination therapy is to combine artemisinin with a longer-acting partner drug to ensure that all parasites are killed and to prevent the emergence of resistance.^3^

In 2008, it became apparent that *P falciparum* was becoming resistant to artemisinin in western Cambodia, and over the next few years resistance was observed in other parts of Cambodia, as well as in Thailand, Vietnam, Myanmar, and Laos.^4^, ^5^, ^6^ Although this finding caused widespread concern, there were some mitigating factors. First, resistance to artemisinin was not complete—ie, artemisinin treatment continued to reduce parasitaemia, albeit at a slower rate than before. Second, partner drugs of artemisinin combination therapy were able to clear parasites despite the slower response of parasites to artemisinin; thus, artemisinin combination therapy remained effective as first-line antimalarial therapy. Third, the spread of resistance was due to multiple emergences of artemisinin resistance, each caused by an independent mutation in the *kelch13* gene and confined to a reasonably small geographical area.^7^, ^8^, ^9^

In 2013, the situation worsened in that artemisinin combination therapy was completely failing to clear parasites in some patients in western Cambodia.^10^ At that time, the form of artemisinin combination therapy used in Cambodia as first-line treatment was dihydroartemisinin–piperaquine and treatment failure was due to increasing resistance to piperaquine. Piperaquine resistance was shown to have a genetic basis, and copy number amplification of the *plasmepsin 2* and *plasmepsin 3* genes was discovered to be a useful genetic marker.^11^, ^12^ Since 2013, the frequency of complete treatment failure in patients receiving dihydroartemisinin–piperaquine has increased rapidly in Cambodia, northeast Thailand, and Vietnam.^13^, ^14^, ^15^, ^16^, ^17^

This spread of dihydroartemisinin–piperaquine treatment failure has been associated with a specific parasite lineage that is spreading across the region.^15^, ^17^ Although the parasites remain sensitive to other forms of artemisinin combination therapy, these reports have caused considerable alarm and debate about whether or not it constitutes a public health emergency.

**Research in context**

**Evidence before the study**

We searched PubMed without language restrictions up to Oct 30, 2017, using the search terms “artemisinin”, “piperaquine”, “multidrug”, “resistance”, and “southeast Asia”. We identified 32 publications about increasing dihydroartemisinin–piperaquine treatment failure and its genetic markers. In two studies, Imwong and colleagues examined dihydroartemisinin–piperaquine treatment failure in Thailand, Laos, and Vietnam, and used microsatellite typing around the *kelch13* gene to show that increasing treatment failure was due to the spread of a particular lineage of artemisinin-resistant parasites originating in western Cambodia.

**Added value of this study**

We did a genome sequence analysis of a large collection of *Plasmodium falciparum* samples from a longitudinal clinical study of antimalarial-drug resistance in western Cambodia. We found that 91% of parasites resistant to dihydroartemisinin–piperaquine were of the KEL1/PLA1 co-lineage, suggesting that they arose from a common epidemiological origin, and that this co-lineage is probably the same lineage observed in other countries by Imwong and colleagues. These data show that the KEL1/PLA1 co-lineage emerged in western Cambodia in the same year that dihydroartemisinin–piperaquine officially became the first-line antimalarial drug, and thereafter spread rapidly for 5 years before the first clinical reports of this major outbreak of multidrug resistance appeared.

**Implications of all the available evidence**

Treatment of *P falciparum* malaria resistant to dihydroartemisinin–piperaquine with alternative antimalarial drug combinations remains possible, but the current outbreak suggests that artemisinin-resistant parasites are gaining increased biological fitness, which might increase the risk of southeast-Asian parasites eventually becoming untreatable and spreading to Africa. Uncertainties can be mitigated by the use of appropriate genetic surveillance technologies to enable malaria control programmes working in the most vulnerable locations to respond as soon as possible to any substantial evolutionary changes in the parasite population. We propose that this strategy should be used as part of the regional malaria elimination policy in southeast Asia.

We did an in-depth genetic analysis of the epidemiological origins and early history of this outbreak using genome sequence data on *P falciparum* samples collected in Cambodia from 2007 to 2013. Our genome sequence data were complemented by open-access sequence data on other samples from southeast Asia generated by the MalariaGEN *Plasmodium falciparum* Community Project.^18^

## Methods

### Genome sequence data

We analysed genome sequence data on 497 *P falciparum* samples collected in clinical studies^6^, ^11^, ^14^, ^19^ of antimalarial drug efficacy in 2010–13 in three provinces of Cambodia where artemisinin and piperaquine resistance was expected to be common (Pursat, western Cambodia), emerging (Preah Vihear, northern Cambodia), or uncommon (Ratanakiri, northeastern Cambodia). Patients were enrolled as part of observational or drug efficacy studies from provincial referral hospitals and district health centres and had presented with uncomplicated *P falciparum* malaria. Details of the sampling protocol, together with enrolment and exclusion criteria, have been reported elsewhere.^6^, ^11^, ^14^, ^19^ All patients provided written informed consent under protocols approved by Cambodia's National Ethics Committee for Health Research and the National Institute of Allergy and Infectious Diseases' institutional review board.

These data were combined with open-access genome sequence data generated by the MalariaGEN *Plasmodium falciparum* Community Project on samples from various locations in southeast Asia, as previously reported.^18^ Information about the contributing studies can be found on the project website. Altogether, this dataset yielded 464 *P falciparum* samples collected in western Cambodia in 2007–13 and 1028 from other locations and timepoints. For all of the samples analysed, sequence data were generated at the Wellcome Sanger Institute with Illumina short-read technology, and genotypes were called with a standardised analysis pipeline.^20^

To minimise the risk of confounding of the multilocus analyses and haplotype analyses by complex infections, we used only samples from south or southeast Asia with sufficient coverage (>75% of the single nucleotide polymorphism [SNP] covered by at least five reads) and with low complexity of infection (within-sample diversity >0·8).

### *Kelch13* genotyping and haplogroup assignment

To analyse mutations in the *P falciparum kelch13* gene that are markers of artemisinin resistance (appendix),^21^ we derived the genotype of *kelch13* for each sample from read counts at non-synonymous SNPs in the propeller and BTB/POZ domains of the kelch13 protein, as described previously.^7^ We reconstructed the probable origin of *kelch13* mutation using chromosome painting.^22^ This method compares haplotypes in a sample to those in the remaining samples and estimates the probability that a genome fragment originates in each of them, while also accounting for recombination and de-novo mutations (appendix).

### Copy number amplifications

We used two orthogonal methods to assess duplication genotypes around *mdr1* and *plasmepsin 2–3*: a coverage-based method and a method based on position and orientation of reads near discovered duplication breakpoints. Details of the methods are described elsewhere.^11^ In short, a coverage-based hidden Markov model was used to identify potential copy number amplifications and their boundaries.^23^ Breakpoints of duplications around *mdr1* and *plasmepsin 2–3* were then identified by visual inspection of soft-clipped reads and paired reads aligned either in the same or the opposite orientation (face-away reads). We then searched all samples for face-away read pairs spanning the breakpoints, and combined results with those of the hidden Markov model. For the *plasmepsin 2–3* locus, breakpoint positions and sequences used in the search are described in the appendix.

### Spread of dihydroartemisinin–piperaquine resistance

We estimated the level of co-ancestry between different locations using chromosome painting. The method was run with the same parameters specified for the *kelch13* haplogroup analysis, but across the whole genome. For each sample, we generated the most likely painting (ie, Viterbi decoding) and aggregated copying vectors according to geographical origin of the donor samples. We then estimated the fraction of the genome copied from each population. Neighbour-joining trees were constructed with the function nj in the R package ape, with default parameters and a genome-wide pairwise genetic distance matrix, as described elsewhere.^18^, ^24^

### Role of the funding source

The funders had no role in study design, data collection, data analysis, data interpretation, or report writing. The corresponding author had full access to all the data in the study and had final responsibility for the decision to submit for publication.

## Results

We analysed genome sequence data on 1492 samples collected at 11 locations across southeast Asia, including 464 samples collected between 2007 and 2013 in Cambodia (table). This collection allowed a detailed longitudinal genetic analysis of the *P falciparum* population of western Cambodia in the period leading up to the first clinical reports of dihydroartemisinin–piperaquine resistance in 2013.

**Table:** Locations of the 1492 samples analysed in this study

		**Total**	**2002–06**	**2007**	**2008**	**2009**	**2010**	**2011**	**2012**	**2013**
South Asia (Bangladesh)		54	0	0	9	11	0	0	34	0
Southeast Asia (west)										
	Myanmar	101	0	0	0	0	0	56	44	1
	Thailand (northwest, south)	333	89	9	83	1	7	39	74	31
Southeast Asia (east)										
	Thailand (northeast)	19	0	0	0	0	0	4	12	3
	Laos	96	0	0	0	0	33	40	23	0
	Vietnam	177	0	0	0	9	57	88	23	0
	Cambodia (northeast)	125	0	0	0	0	36	59	20	10
	Cambodia (north)	123	0	0	0	0	0	61	41	21
	Cambodia (west)	464	0	25	41	58	102	141	59	38

**Figure 1:**
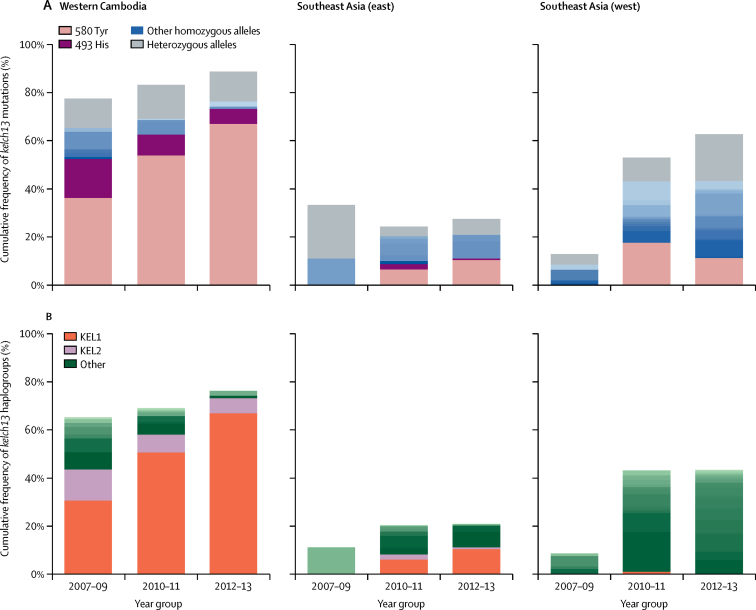
Cumulative frequency of *kelch13* mutations (A) and haplogroups (B) Southeast Asia (east) includes northern and northeastern Cambodia, Vietnam, Laos, and northeastern Thailand. Southeast Asia (west) includes northwestern and southern Thailand and Myanmar.

689 (46%) of 1492 samples carried *kelch13* mutations, including 136 (9%) samples that were heterozygous because of mixed infection (appendix). We observed 24 distinct *kelch13* mutations, each denoted by their effect on the aminoacid sequence (figure 1; appendix). In the 553 homozygous samples with *kelch13* mutations, the most frequent alleles were 580Tyr (n=317), 493 His (n=56), 539Thr (n=43), 543Thr (n=24), 441Leu (n=16), 561His (n=15), and 675Val (n=15).

*Kelch13* mutations were observed throughout the entire geographical region, with the exception of Bangladesh, and increased in frequency over time (figure 1). Western Cambodia had an extremely high prevalence of *kelch13* mutations (384 [83%] of 464 samples, including 61 [13%] heterozygous samples), and their frequency increased from 40% (ten of 25) in 2007 to 84% (32 of 38) in 2013. Of 323 samples from western Cambodia with homozygous *kelch13* mutations, 241 (75%) were carrying the 580Tyr allele (appendix).

The observation of 24 different *kelch13* mutations suggested that at least 24 epidemiological origins of artemisinin resistance existed in the sampled locations. However, some *kelch13* mutations have multiple independent origins, so that two samples with the same mutation might not be epidemiologically related (ie, their alleles are identical by state, but they might not be identical by descent).^7^, ^8^ To address this question, we analysed the haplotype structure of *kelch13* and its flanking regions in all 553 samples with homozygous mutations and used statistical chromosome painting to estimate the level of shared ancestry between samples.^22^ This approach allowed assignment of each sample to a *kelch13* haplogroup, defined here as a group of samples with the same *kelch13* mutation and strong haplotypic similarities indicative of recent shared lineage at the *kelch13* locus (appendix). This approach identified 38 *kelch13* haplogroups, each of which was presumed to represent a distinct lineage of artemisinin resistance (appendix). The 580Tyr allele was found in six different *kelch13* haplogroups, of which the most common was the KEL1 lineage (appendix).

The KEL1 lineage accounted for 266 (48%) of 553 artemisinin-resistant samples in this dataset. The lineage was predominantly found in western Cambodia, but was also observed in other parts of Cambodia, as well as in Vietnam and Laos, where its genetic background appeared to be different (figure 1; appendix). In western Cambodia, the KEL1 lineage was at 4% (one of 25) frequency in 2007, which increased to 63% (24 of 38) in 2013. The lineage appears to have spread through the parasite population by recombination—ie, it is found in parasites that are considerably different at the whole-genome level, as can be visualised with a genome-wide neighbour-joining tree (appendix).

**Figure 2:**
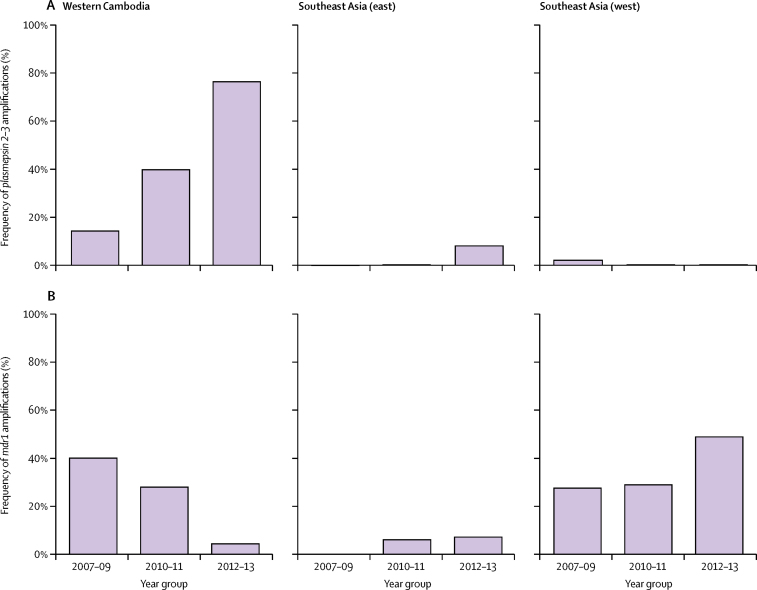
Frequency of *plasmepsin 2–3* (A) and *mdr1* (B) amplifications Southeast Asia (east) includes northern and northeastern Cambodia, Vietnam, Laos, and northeastern Thailand. Southeast Asia (west) includes northwestern and southern Thailand and Myanmar.

Amplifications of *plasmepsin 2–3*, which are markers of piperaquine resistance, were observed in 185 (41%) of 456 samples from western Cambodia and in 14 (1%) of 1009 samples from elsewhere (calls were inconclusive for 27 samples; figure 2; appendix). Two genetic features of the *plasmepsin 2–3* amplifications indicated that they mostly had the same epidemiological origin. First, the breakpoint sequences of the amplification (ie, the sequences around the point where the additional copy of the gene is inserted) were identical in all but six samples (appendix). Second, most samples carrying the amplification had highly similar haplotypes surrounding the amplified genes, which were different from those without the amplification (appendix). Based on haplotype analysis, 186 (93%) of 199 samples with the amplification had strong evidence of recent shared ancestry (fewer than five differences out of 1454 SNPs with minor allele frequency of >1%), and 11 (6%) samples could plausibly have arisen from the same recent ancestor after allowing for recombination or mixed infections. We refer to this lineage as PLA1. The remaining two samples with the amplification were from northwest Thailand and had a substantially different haplotype.

Amplifications of *plasmepsin 2–3* were already circulating at low frequency in 2002–03.^12^ However, in this dataset, *plasmepsin 2–3* amplifications were absent in 2007 but then rapidly increased to 79% (30/38) frequency in western Cambodia by 2013. This finding differs from the Thailand–Myanmar border, where only two amplifications were observed in 2008 and none after that timepoint. In northern Cambodia, *plasmepsin 2–3* amplifications were first observed in 11 samples collected in 2012 and 2013.

The combination of *kelch13* mutation and *plasmepsin 2–3* amplification is a marker of dihydroartemisinin–piperaquine treatment failure.^11^ By analysing this combination of markers, we identified 154 samples resistant to dihydroartemisinin–piperaquine, after exclusion of samples with heterozygous genotypes, which were unsuitable for this analysis. We found that 145 (94%) samples resistant to dihydroartemisinin–piperaquine carried the *kelch13* 580Tyr allele and 140 (91%) belonged to the KEL1 lineage. Six (4%) resistant samples carried the 493His allele and belonged to the KEL2 lineage (appendix). We identified 15 samples from western Cambodia and two from Thailand that carried *plasmepsin 2–3* amplifications, but without *kelch13* mutation.

**Figure 3:**
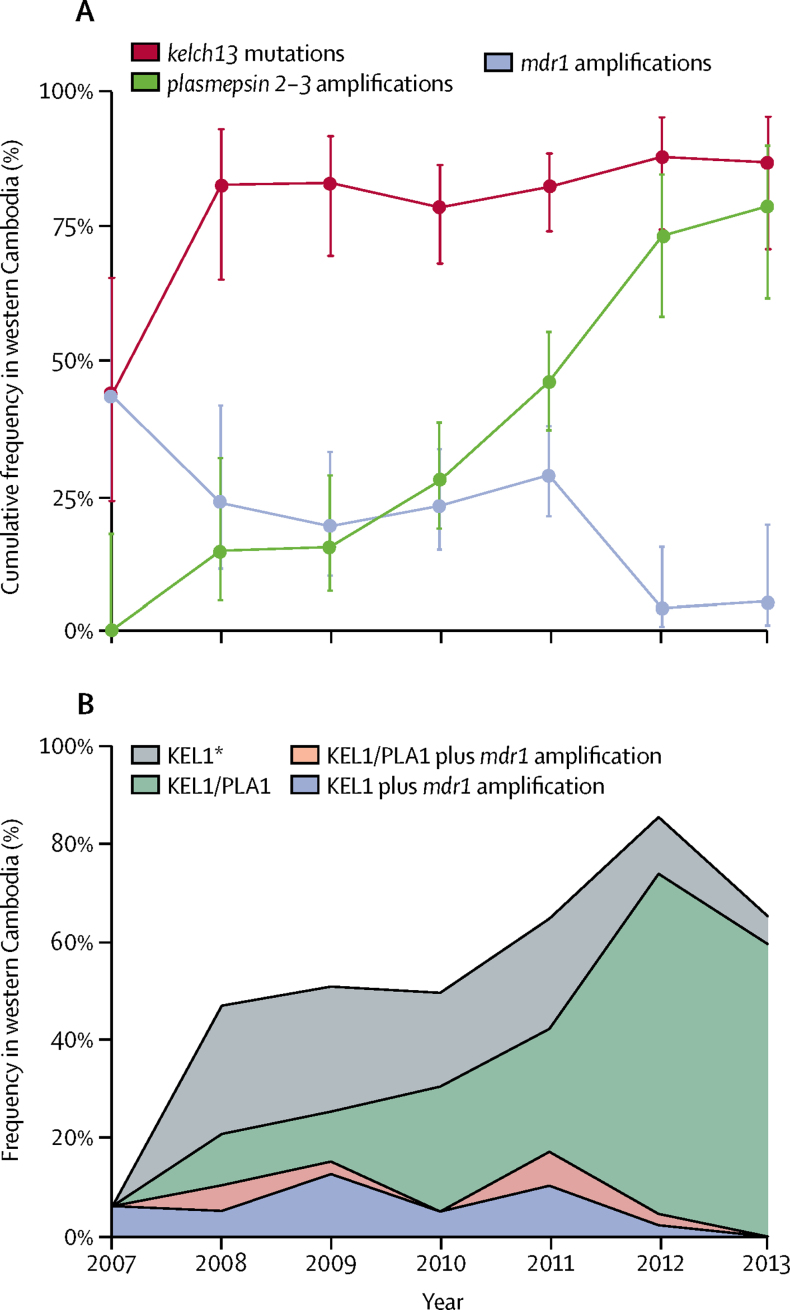
Frequency in western Cambodia of molecular markers for the three most commonly used drugs in the area and of the KEL1 lineage from 2007 to 2013 (A) Cumulative frequency over time of *kelch13* mutations, *plasmepsin 2–3* amplifications, and *mdr1* amplifications; error bars are 95% CIs of the frequencies, based on sample sizes. (B) Frequency over time of the dominant haplogroup KEL1; 22 samples in which the presence of either *plasmepsin 2–3* or *mdr1* amplifications could not be established reliably were excluded from the graph. *These parasites had only single copies of *plasmepsin 2–3* and *mdr1*.

These data indicate that resistance to dihydroartemisinin–piperaquine was present in western Cambodia as early as 2008 and then rapidly increased in frequency, from 16% (five of 31) in 2008 to 68% (25 of 37) in 2013 (figure 3). From the time of its emergence, resistance to dihydroartemisinin–piperaquine was associated with the KEL1 lineage, which was observed in eight (80%) of ten resistant samples obtained from western Cambodia in 2008 and in 55 (93%) of 59 resistant samples obtained from western Cambodia in 2012–13. In the same period, the frequency of KEL1 samples carrying *plasmepsin 2–3* amplifications (all of the PLA1 lineage) increased in western Cambodia, from 31% (four of 13) in 2008 to 92% (22 of 24) in 2013.

The acquisition of *plasmepsin 2–3* amplifications by parasites of the KEL2 lineage (carrying the *kelch13* 493His resistance allele) appears to be more recent, and these parasites account for six (6%) of the 105 samples resistant to dihydroartemisinin–piperaquine in western Cambodia between 2011 and 2013 (appendix). Genomic evidence suggests that these amplifications are of the PLA1 lineage (appendix); thus, this acquisition probably occurred through introgression via recombination, possibly with parasites belonging to the KEL1 lineage.

Based on these genetic data, resistance to dihydroartemisinin–piperaquine appears to have been confined to western Cambodia until 2011, but in 2012–13 it was observed in 11 samples from northern Cambodia and in one sample from Laos close to the northern Cambodian border. Analysis of the *kelch13* and *plasmepsin 2–3* loci in these northern Cambodian samples showed that they resembled most of the resistant samples from western Cambodia, suggesting they all belonged to the KEL1/PLA1 co-lineage. This finding suggests that resistance to dihydroartemisinin–piperaquine has spread from western to northern Cambodia.

**Figure 4:**
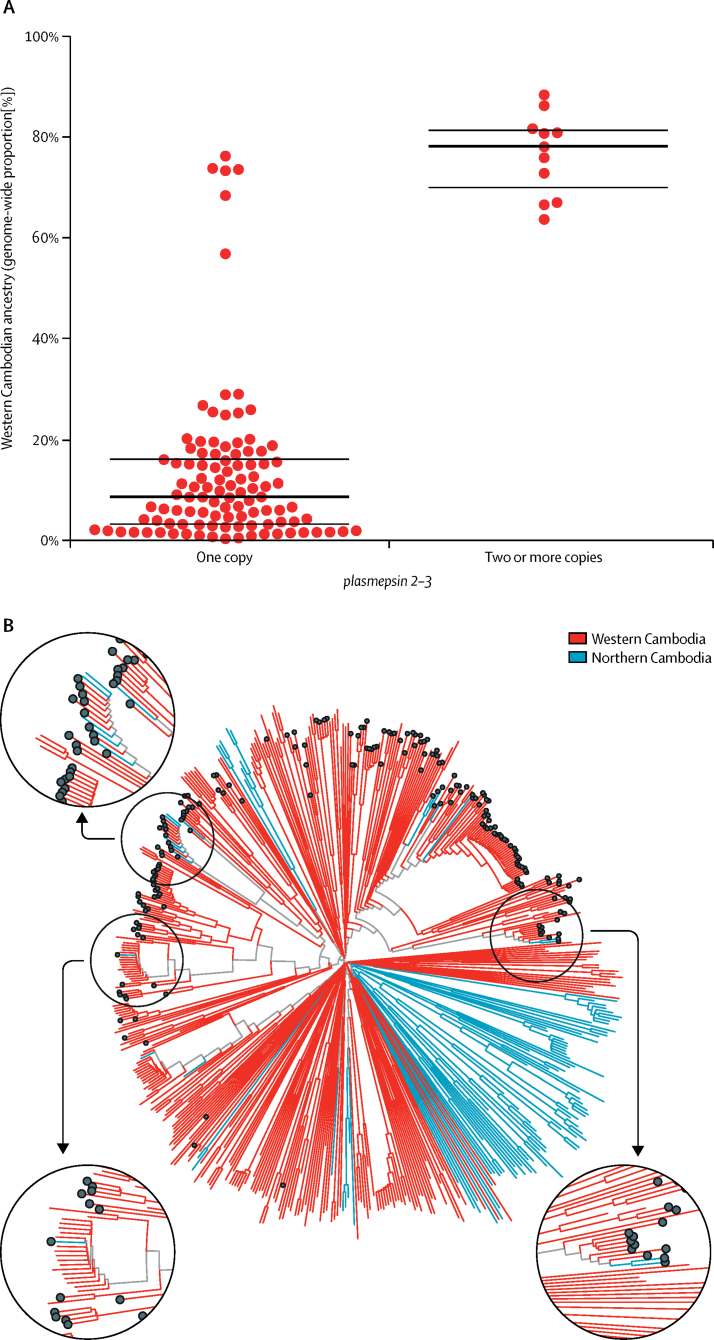
Spread of dihydroartemisinin–piperaquine resistance to north Cambodia (A) Each point represents a sample from northern Cambodia; bold lines indicate medians and thin lines indicate IQRs. (B) Genome-wide neighbour-joining tree of all samples from northern and western Cambodia in the dataset, with those carrying *plasmepsin 2-3* amplifications identified by black dots at the tip. The circular subpanels show a magnified view of parts of the tree containing samples from northern Cambodia carrying *plasmepsin 2–3* amplifications.

To examine this question in more detail, we did chromosome painting across the whole genome and estimated co-ancestry between samples from different geographical locations. We found that, in northern Cambodia, samples with markers of dihydroartemisinin–piperaquine resistance had much higher levels of western Cambodian co-ancestry than the rest of the population (figure 4A). Construction of a neighbour-joining tree with whole-genome data showed that samples from northern Cambodia with markers of dihydroartemisinin–piperaquine resistance grouped closely with parasites from western Cambodia, rather than with other parasites from northern Cambodia (figure 4B). Taken together, these findings indicate that emergence of dihydroartemisinin–piperaquine resistance in northern Cambodia in 2012 was the result of migration of parasites from western Cambodia.

Artesunate–mefloquine was the first-line artemisinin combination therapy in Cambodia before the initial use of dihydroartemisinin–piperaquine in 2008, and has continued to be widely used in other parts of southeast Asia.^25^ Increasing piperaquine resistance in western Cambodia was accompanied by a reduction in mefloquine resistance.^14^ We explored this association by analysing *mdr1* amplifications that are known markers of mefloquine resistance.^26^ Between 2007 and 2013, the frequency of *mdr1* amplifications in western Cambodia decreased from 59% (ten of 17) to 6% (two of 36), by contrast with Myanmar and Thailand, where the frequency increased from 13% (one of eight) in 2007 to 63% (19 of 30) in 2013. This decline in *mdr1* amplifications in western Cambodia was directly associated with the evolution and expansion of parasites belonging to the KEL1 haplogroup (figure 3). As the KEL1 lineage increased in frequency, it progressively lost *mdr1* amplifications and gained *plasmepsin 2–3* amplifications. By 2013, when the KEL1 lineage had reached a frequency of 65% (24/37) in western Cambodia, 92% (22/24) of the KEL1 samples had *plasmepsin 2–3* amplifications, whereas none had *mdr1* amplifications.

## Discussion

Whether the rapid spread of resistance to dihydroartemisinin–piperaquine in southeast Asia will derail malaria control worldwide is highly debated.^15^, ^17^, ^27^, ^28^ WHO has determined that it is not a public health emergency, but this view is challenged by some malaria experts.^27^, ^28^ The findings of this study shed light on four questions pertinent to the ongoing debate: what is the nature of the parasites that are causing the problem? How rapidly have they spread? How are they likely to respond to alternative drug combinations? What are the major risks and uncertainties in the long term?

First, in terms of population genetics, what are the essential features of the resistant parasites? We found that 91% of the samples carried a *kelch13* 580Tyr allele of the KEL1 lineage and 4% carried a *kelch13* 493His allele of the KEL2 lineage. Thus, most but not all of the parasites resistant to dihydroartemisinin–piperaquine acquired artemisinin resistance from a single epidemiological origin, despite identification of 38 putative independent origins of artemisinin resistance in our study.

At the *plasmepsin 2–3* locus, we identified only two epidemiological origins of gene amplifications associated with piperaquine resistance. One of these was reasonably rare, being found in only two parasites from Thailand. All of the parasites resistant to dihydroartemisinin–piperaquine belonged to a single *plasmepsin 2–3* lineage that we refer to as PLA1.

The genetic features of the KEL1/PLA1 co-lineage are concordant with reports of parasites resistant to dihydroartemisinin–piperaquine in Thailand, Laos, and Vietnam.^15^, ^17^ Imwong and colleagues^15^, ^17^ refer to these parasites as the PfPailin lineage, suggesting that this lineage of *P falciparum* is uniform, analogous to a viral or bacterial strain. However, malaria parasites, unlike viruses and bacteria, undergo sexual recombination with each transmission cycle and, therefore, this nomenclature is potentially misleading. Our data show that the KEL1 lineage of artemisinin resistance is carried by parasites of diverse genetic backgrounds and, although parasites of the KEL1/PLA1 co-lineage have high levels of shared ancestry relative to the general parasite population, they are not genetically homogeneous. Additionally, the KEL1 and PLA1 lineages are separate and possibly emerged independently. This concept is central to understanding how multidrug-resistant parasites might evolve in the future—ie, through recombination with other parasites and incorporation of new genetic features.

Second, when did this group of parasites emerge and how rapidly have they spread? Our data show that parasites in the KEL1 lineage were present at low frequencies in western Cambodia as early as 2007. At that time, they carried *mdr1* amplifications that confer resistance to mefloquine and did not carry *plasmepsin 2–3* amplifications. The KEL1/PLA1 co-lineage was first observed in 2008, the same year that dihydroartemisinin–piperaquine was introduced as the first-line antimalarial drug in western Cambodia. Nevertheless, piperaquine has been extensively used in western Cambodia, as a monotherapy in the 1990s and then in combination with artemisinin in the late 2000s. In neighbouring provinces, *plasmepsin 2–3* amplifications were circulating at low frequencies in 2002–03, but had increased in frequency by 2008.^12^ Thus, although we did not find *plasmepsin 2–3* amplifications in this dataset in 2007, it is possible that PLA1 existed before at a low frequency, independently of KEL1. Over the time period and in the locations surveyed here, PLA1 was mainly linked to KEL1, but it was also seen in parasites with the KEL2 haplogroup and in those without *kelch13* mutations.

Before the emergence of dihydroartemisinin–piperaquine resistance, the frequency of artemisinin-resistant parasites in western Cambodia was already high, but they comprised a diverse set of *kelch13* mutations arising from multiple epidemiological origins, each of which tended to remain fairly localised. After the KEL1/PLA1 co-lineage emerged in 2008, it spread rapidly and extensively across western Cambodia, reaching a frequency of more than 60% in the parasite population. Our data show that the KEL1/PLA1 co-lineage appeared in northern Cambodia in 2012, probably due to the spread of parasites from western Cambodia. Although the genetic typing methods used by Imwong and colleagues^15^, ^17^ to characterise *kelch13* haplotypes and *plasmepsin 2–3* amplifications were different from those used in this study, it seems likely from their data that the parasites resistant to dihydroartemisinin–piperaquine that appeared in northeastern Thailand and Laos in 2014–15, and in Vietnam in 2016, correspond to the KEL1/PLA1 co-lineage.

Third, what short-term predictions can be made about the likely response of the resistant parasites to alternative drug combinations? The KEL1 lineage was initially associated with *mdr1* amplifications that are markers of mefloquine resistance, but this association has lessened over time, and the most recent KEL1/PLA1 samples in this dataset do not carry *mdr1* amplifications. Therefore, at present, parasites resistant to dihydroartemisinin–piperaquine are sensitive to mefloquine, and artesunate–mefloquine is now being used successfully as first-line antimalarial treatment in Cambodia. Evidence also suggests that parasites resistant to dihydroartemisinin–piperaquine are responsive to artesunate–pyronaridine, a new artemisinin combination therapy.^27^

It is reasonable to expect that the switch to artesunate–mefloquine in Cambodia will cause piperaquine resistance to decline, but this switch might also cause mefloquine resistance to rise, particularly because KEL1 parasites evidently have no problem in switching between mefloquine and piperaquine resistance. Evidence suggests that a negative interaction exists between *mdr1* and *plasmepsin 2–3* amplifications; they rarely co-occur in samples from Cambodia.^11^, ^12^ Further studies are needed to assess whether this negative association is the result of a natural antagonism, in which the use of piperaquine decreases mefloquine resistance and vice versa. If that is the case, the effectiveness of artemisinin combination therapy in southeast Asia could be maintained by use of mefloquine and piperaquine in combination or in rotation. However, any strategy will need to be closely monitored because it has the potential to cause emergence of joint resistance to both drugs.

Finally, what are the long-term risks and how might the level of uncertainty be reduced? The main risks are that *P falciparum* malaria will eventually become untreatable in southeast Asia, and that this resistance will spread to Africa. Many parallels exist between drug resistance outbreaks and cancer. In cancer diagnosis, the term aggressive means a tumour that is rapidly spreading, as opposed to one that remains localised. By this definition, the KEL1/PLA1 co-lineage can be described as an aggressively spreading form of drug resistance.

The rapid spread of KEL1/PLA1 suggests that artemisinin-resistant parasites are acquiring increased biological fitness, and to what extent this increased biological fitness increases the risk of partner-drug failure and transcontinental spread is unknown. On one hand, the risk might be fairly low; the success of the KEL1 lineage might have been simply due to its association with PLA1 at a time when dihydroartemisinin–piperaquine was the first-line antimalarial. In this case, spread of resistance could potentially be contained through strategic management of dihydroartemisinin–piperaquine use. On the other hand, the aggressive spread of KEL1 might have been due to factors that are independent of its association with PLA1. KEL1 carries the 580Tyr allele, which has more independent origins than any other artemisinin resistance allele. The frequency of the 580Tyr allele is much higher than the frequency of other artemisinin resistance alleles, not only in western Cambodia but also on the Thailand–Myanmar border.^29^ Given that the allele has emerged independently in these two locations, it might have superior fitness that is independent of its genetic background. It is also possible that the KEL1 lineage is progressively undergoing evolutionary adaptation by becoming linked to compensatory or synergistic mutations in other genes that act to increase its transmissibility and biological fitness.

Malaria policy makers now face a dilemma. On one hand, malaria remains treatable, and its prevalence has been sufficiently reduced in Cambodia and neighbouring countries that regional malaria elimination seems feasible. On the other hand, the situation is extremely fragile, and there is considerable uncertainty about how the parasite population will evolve in response to the next round of interventions. Our data show that the KEL1/PLA1 co-lineage emerged in 2008, the same year that dihydroartemisinin–piperaquine officially became the first-line antimalarial in western Cambodia, and then spread rapidly for 5 years before the first clinical reports emerged. It would be catastrophic if the same were to happen for the last remaining antimalarials that are effective in southeast Asia. However, unlike the situation in 2008, provision of national malaria control programmes, with tools for high-resolution genetic surveillance of all observed malaria cases in the most vulnerable locations, is now technically feasible. We propose that this strategy should be used as part of the regional malaria elimination policy because it would enable malaria control programmes to respond as soon as possible to evolutionary changes in the parasite population, and thereby reduce uncertainty and risk. We also suggest that these data should be made openly available, so that all countries in the region and the international malaria community can cooperate to find a solution if a particular intervention strategy starts to fail. With use of appropriate technologies and concerted action, major outbreaks of resistance should not go unnoticed in the future and the risk of a global health emergency should be reduced.
