# Supplementary material for: Origins of the current outbreak of multidrug-resistant malaria in southeast Asia: a retrospective genetic study
Source: Lancet Infect Dis. 2018 Mar;18(3):337–45. doi: 10.1016/S1473-3099(18)30068-9 (PMC5835763; doi:10.1016/S1473-3099(18)30068-9)
Supplement: Supplementary appendix [file mmc1.pdf]

THE LANCET Infectious Diseases

Supplementary webappendix

This webappendix formed part of the original submission and has been peer reviewed. We post it as supplied by the authors.

Supplement to: Amato R, Pearson R D, Almagro-Garcia J, et al. Origins of the current outbreak of multidrug-resistant malaria in southeast Asia: a retrospective genetic study. *Lancet Infect Dis* 2018; published online Feb 1. http://dx.doi.org/10.1016/S1473-3099(18)30068-9.

Appendix

Origins of the current outbreak of multidrug resistant malaria in Southeast Asia: a retrospective genetic study

Roberto Amato PhD, Richard D. Pearson PhD, Jacob Almagro-Garcia PhD, Chanaki Amaratunga PhD, Pharath Lim MD, Seila Suon MD, Sokunthea Sreng, Eleanor Drury BSc[Hons], Jim Stalker MA, Olivo Miotto PhD, Rick M. Fairhurst MD, and Dominic P. Kwiatkowski FRCP

Contents

Number of *kelch13* wild-type, heterozygous, and mutant samples in the dataset 2

Geographical distribution of the 38 *kelch13* haplogroups 3

Temporal distribution of the 38 *kelch13* haplogroups 4

Temporal and geographical distribution of samples carrying *plasmepsin 2-3* amplification ...5

Distribution of samples carrying *plasmepsin 2-3* amplification across the *kelch13* haplogroups 6

Co-ancestry matrix of 553 samples carrying homozygous *kelch13* mutations 7

Haplotype analysis of samples carrying *kelch13* mutations 8

Genetic relatedness of KEL1 samples to all other samples 9

Analyses of haplotypes surrounding the *plasmepsin 2-3* genes 10

Reconstruction of *kelch13* mutations epidemiological origin 11

Breakpoints identified for the *plasmepsin 2-3* amplification 12

Alleles that characterize the KEL1 haplogroup 13

Supplementary Table 1. Number of *kelch13* wild-type, heterozygous, and mutant samples in the dataset. For each mutation, we also report the level of validation (validated, candidate, associated, unknown) of the allele as an artemisinin resistance marker, as specified by the WHO guidelines (http://apps.who.int/iris/bitstream/10665/255213/1/WHO-HTM-GMP-2017.9-eng.pdf?ua=1 - accessed 30/Nov/17). SAS = South Asia; WSEA = Southeast Asia - West; ESEA = Southeast Asia - East.

			SAS	WSEA		ESEA						
kelch13	***kelch13* mutation**	**Level of validation as resistance marker**	Bangladesh	Myanmar	Thailand (Northwest and South)	Cambodia (North)	Cambodia (Northeast)	Cambodia (West)	Vietnam	Thailand (Northeast)	Laos	**Total**
Wild-type			53	60	207	86	121	80	103	1	92	**803**
Het			1	7	40	4	1	61	17	3	2	**136**
Mutant	*Total*			34	86	33	3	323	57	15	2	**553**
	580Y	Validated		11	24	26	3	241	9	3		317
	493H	Validated				5		47	4			56
	539T	Validated				2		23	4	12	2	43
	543T	Validated						2	22			24
	441L	Candidate		5	11							16
	561H	Candidate		2	13							15
	675V	Candidate		2	13							15
	553L	Candidate			2				9			11
	538V	Candidate			8							8
	449A	Candidate		2	3			2				7
	574L	Candidate		6	1							7
	353Y	Unknown							5			5
	527H	Unknown			5							5
	568G	Candidate							4			4
	481V	Associated			2			2				4
	446I	Candidate		3								3
	719N	Associated						3				3
	673I	Associated		2								2
	584V	Associated						2				2
	438N	Unknown		1	1							2
	443S	Unknown			1							1
	395Y	Unknown						1				1
	614L	Unknown			1							1
	537I	Associated			1							1

Supplementary Table 2. Geographical distribution of the 38 *kelch13* haplogroups.

kelch13 haplogroup	Samples	*kelch13* mutation	Cambodia (West)	Cambodia (North)	Cambodia (Northeast)	Laos	Thailand (Northeast)	Vietnam	Myanmar	Thailand (Northwest and South)
**KEL1**	266	580Y	226	26	2		2	9		1
**KEL2**	49	493H	40	5				4		
**KEL3**	40	539T	21	2		2	11	4		
**KEL4**	28	580Y			1		1		11	15
**KEL5**	24	543T	2					22		
**KEL6**	15	675V							2	13
**KEL7**	13	580Y	13							
**KEL8**	11	561H								11
**KEL9**	9	553L						7		2
**KEL10**	8	580Y								8
**KEL11**	8	538V								8
**KEL12**	8	441L								8
**KEL13**	7	574L							6	1
**KEL14**	7	449A	2						2	3
**KEL15**	5	441L							3	2
**KEL16**	5	527H								5
**KEL17**	4	493H	4							
**KEL18**	4	568G						4		
**KEL19**	4	353Y						4		
**KEL20**	4	561H							2	2
**KEL21**	4	481V	2							2
**KEL22**	3	493H	3							
**KEL23**	3	441L							2	1
**KEL24**	3	719N	3							
**KEL25**	3	446I							3	
**KEL26**	2	553L						2		
**KEL27**	2	539T	1				1			
**KEL28**	2	438N							1	1
**KEL29**	2	584V	2							
**KEL30**	2	673I							2	
**KEL31**	1	580Y	1							
**KEL32**	1	614L								1
**KEL33**	1	353Y						1		
**KEL34**	1	580Y	1							
**KEL35**	1	537I								1
**KEL36**	1	395Y	1							
**KEL37**	1	539T	1							
**KEL38**	1	443S								1

Supplementary Table 3. Temporal distribution of the 38 *kelch13* haplogroups.

kelch13 haplogroup	2002-2006	2007	2008	2009	2010	2011	2012	2013
KEL1		1	14	23	46	101	48	33
KEL2		6	2	8	14	12	2	5
KEL3		2	5	2	4	16	8	3
KEL4						19	8	1
KEL5					7	13	4	
KEL6			2			8	5	
KEL7			3	4	5	1		
KEL8							5	6
KEL9					4	4	1	
KEL10							4	4
KEL11						1	7	
KEL12			1			1	3	3
KEL13						5	2	
KEL14				2			5	
KEL15						2	2	1
KEL16			4		1			
KEL17		1	1	2				
KEL18					3	1		
KEL19					4			
KEL20	2						2	
KEL21			2			1		1
KEL22					2	1		
KEL23						2	1	
KEL24						1		2
KEL25						2	1	
KEL26				1	1			
KEL27						1	1	
KEL28			1				1	
KEL29			1	1				
KEL30						2		
KEL31						1		
KEL32							1	
KEL33					1			
KEL34						1		
KEL35							1	
KEL36				1				
KEL37						1		
KEL38	1							

Supplementary Table 4. Temporal and geographical distribution of samples carrying *plasmepsin 2-3* amplification.

	Cambodia (West)	Cambodia (North)	Cambodia (Northeast)	Thailand (Northwest)
2008	8			2
2009	9			
2010	31			
2011	64			
2012	43	4		
2013	30	7	1	
Total	**185**	**11**	**1**	**2**

Supplementary Table 5. Distribution of samples carrying *plasmepsin 2-3* amplification across the *kelch13* haplogroups.

kelch13	***kelch13* haplogroup**	**2008**	**2009**	**2010**	**2011**	**2012**	**2013**	**Total**
Wild-type		2			10	1	4	**17**
Het		3	1	7	8	8	1	**28**
Mutant	*Total*	5	8	24	46	38	33	**154**
	KEL1	4	6	22	41	37	30	**140**
	KEL2				3	1	2	**6**
	KEL7	1	1	1	1			**4**
	KEL22			1				**1**
	KEL24						1	**1**
	KEL31				1			**1**
	KEL36		1					**1**

Supplementary Figure 1. Co-ancestry matrix of 553 samples carrying homozygous *kelch13* mutations. (A) The matrix has 553 rows and columns and is symmetric along the diagonal. The complete hierarchical clustering dendrogram is reported on the left. Red colours represent higher level of co-ancestry (arbitrary unit). (B) Two-dimensional visualization of the same co-ancestry matrix using t-SNE, a dimensionality reduction approach. Each dot represents a sample coloured according the to the *kelch13* haplogroup it belongs to, using the same colour scheme as Figure 1. Yellow and pink samples carry KEL1 and KEL2 haplogroups, respectively. Ellipses capture 90% of the spread of each haplogroup.

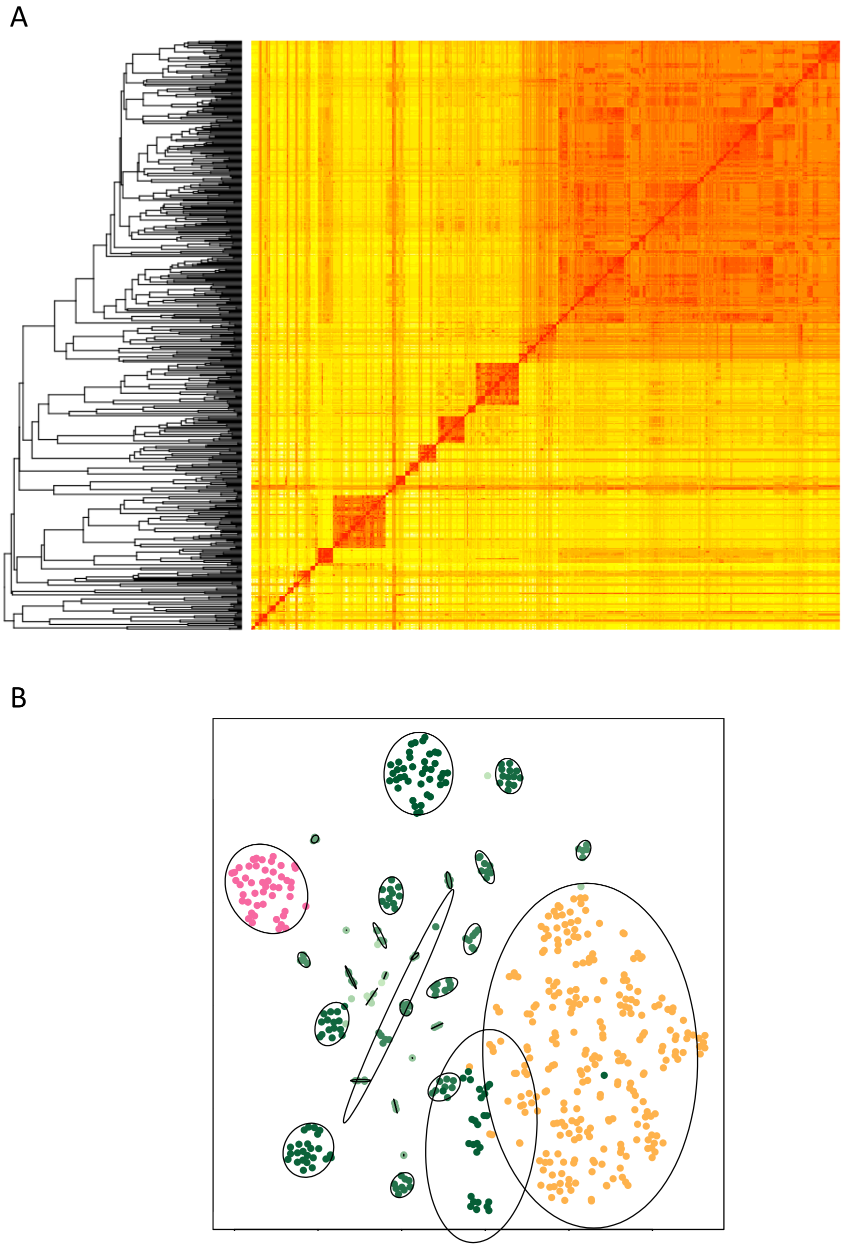

Supplementary Figure 2. Haplotype analysis of samples carrying *kelch13* mutations. Haplotype diagram where each vertical column represents a SNP (MAF >1%) within approximately 100 kbp either side of the *kelch13* gene, and each horizontal line a sample; at the intersection, black lines represents a non-reference genotype allele in the sample (a read majority call is used for heterozygous genotypes). Grey line at the bottom reports the position of the SNPs within chromosome 13, with the *kelch13* genes highlighted in red. A complete hierarchical clustering dendrogram is reported on the left, based on the co-ancestry matrix calculated using statistical chromosome painting. Bold black lines join clusters whose distance is above the cut-off point (height=14) while thin grey lines show the internal structure of each cluster. Highlighted in orange and red are samples belonging to KEL1 and KEL2, respectively.

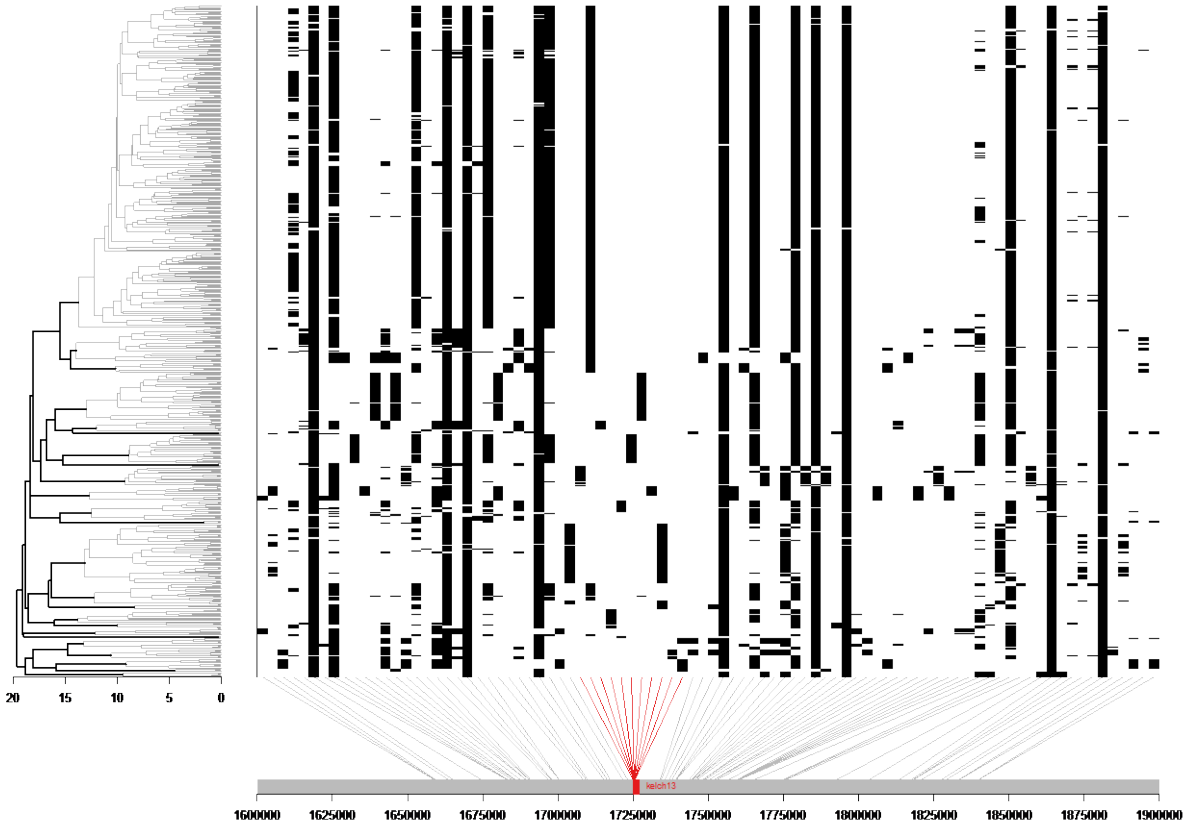

Supplementary Figure 3. Genetic relatedness of KEL1 samples to all other samples. Genome-wide neighbour-joining tree of all samples in the dataset based on their overall genetic similarity. Each segment represents one sample and is coloured according to the region of collection. Parasites carrying the dominant haplogroup KEL1 are identified by a black dot at the tip.

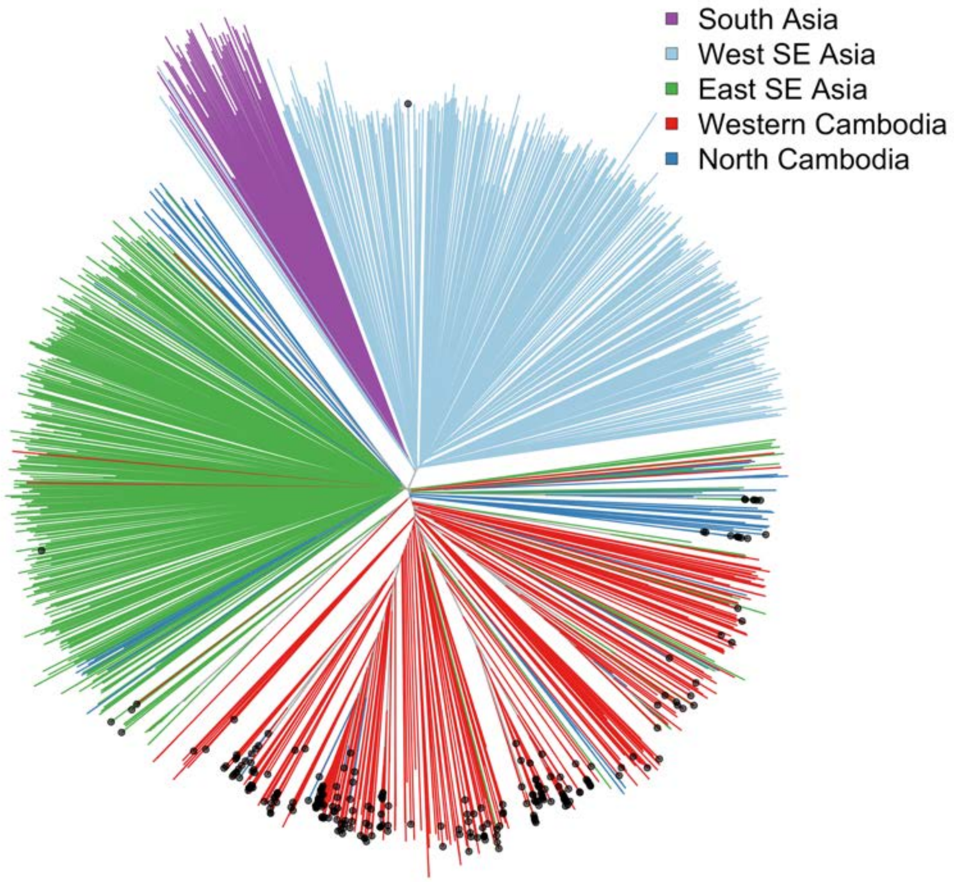

Supplementary Figure 4. Analyses of haplotypes surrounding the *plasmepsin 2-3* genes. Haplotype diagram where each vertical column represents a SNP (MAF >1%) within 100 kbp around the *plasmepsin 2-3* genes, and each horizontal line a sample; at the intersection, black lines represents a non-reference genotype allele in the sample (a read majority call is used for heterozygous genotypes). Haplotypes are shown for samples carrying the amplification (top, n=199) and wild-type alleles (bottom, n=1266); 26 unclassified samples are not shown. The first column of coloured lines on the left (P23A) reports the presence of the amplification and the set of breakpoints identified (black, red, or green for each one of the three sets of breakpoints, white for wild-type). The second column reports the origin of the sample for the three major regions where amplifications are observed (WKH = western Cambodia, red; NKH = northern Cambodia, blue; WTH = northwestern Thailand, green; white = elsewhere). The last column reports the two major *kelch13* haplogroups associated with the amplification (KEL 1 = orange; KEL2 = purple). Grey line at the bottom reports the position of the SNPs within chromosome 14, with the *plasmepsin 2-3* genes highlighted in red. A complete hierarchical clustering dendrogram is reported on the left, with mutant and wild-type samples clustered independently for clarity. The height of the joints in the dendrogram reports the maximum number of differences between any two haplotypes.

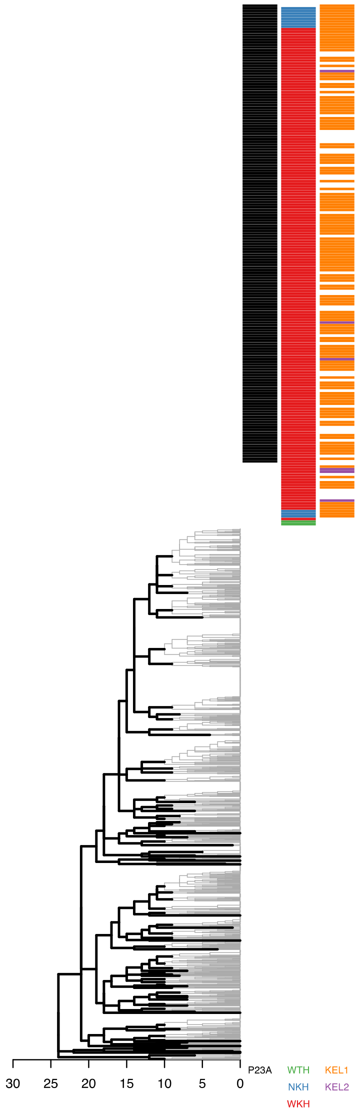

Supplementary Note 1. Reconstruction of kelch13 mutations epidemiological origin.

We reconstructed the probable origin of *kelch13* mutation using chromosome painting.[1] This method compares haplotypes in a sample to those in the remaining samples, and estimates the probability that a genome fragment originates in each of them while also accounting for recombination and *de novo* mutations.

For all and only *kelch13* homozygous mutant samples (n=553), we ran chromosome painting on the entire chromosome 13, obtaining posterior copying probabilities for all loci (an approximation of the probability of two samples being closest neighbours, for each locus, in the underlying genealogy). Mutation rate (i.e. miscopying parameter) was estimated per sample using the Watterson's estimator, and we assumed a uniform recombination map with a rate of 500 kbp/cM. The scaling parameter (termed effective population size) was set to 1000. To account for the presence of residual heterozygous genotypes due to mixed infections, ε (the probability of emitting a mixed call) was set to 10^{-8}.[2] We repeated the analysis varying this parameter set, to assess the effects of misspecification, and results were found to be very similar qualitatively (data not shown). We aggregated these probabilities by taking, for each sample, their average inside the boundaries of the *kelch13* gene (Pf3D7_13_v3: 1724817-1726997). Different aggregation methods, including utilising single variants inside the gene, produced identical results (data not shown). This process resulted in one "copying vector" k_i of 553 elements per sample i $(i = 1, \ldots, 553)$ reporting the probability of that sample being close to all remaining ones in the underlying genealogy of the *kelch13* locus.

To assign samples to haplogroups, these copying vectors k_i were assembled together to form a matrix K of dimension 553×553, which can be interpreted as a measure of ancestral similarity between all pairs of samples. We performed a complete hierarchical clustering using as distance matrix the complement of $\log(K + K^T)$ (Appendix, page 7). This analysis was performed using the function `hclust(method="complete")` as implemented in R version 3.3.2.[3] The resulting dendrogram was subsequently cut to produce discreet clusters. The cut-off was determined heuristically in order to maximise cluster homogeneity by visually inspecting: (i) the haplotype structure (Appendix, page 8); (ii) the distance matrix (Appendix, page 7); and (iii) a t-SNE reduction of the distance matrix (Appendix, page 7). This process identified 24 distinct clusters. The t-SNE representation was calculated using the R function and package `tsne` and ran with default parameters.[4] Finally, haplogroups were defined as groups of samples having the same *kelch13* mutation and belonging to the same cluster.

References:

1 Lawson DJ, Hellenthal G, Myers S, Falush D. Inference of population structure using dense haplotype data. *PLoS Genet* 2012; **8**: e1002453.

2 MalariaGEN Plasmodium falciparum Community Project. Genomic epidemiology of artemisinin resistant malaria. *Elife* 2016; **5**: 1043–9.

3 R Development Core Team. R: A Language and Environment for Statistical Computing. Vienna, Austria, 2009 http://www.r-project.org.

4 Donaldson J. tsne: T-distributed Stochastic Neighbor Embedding for R (t-SNE). 2012. https://cran.r-project.org/package=tsne.

Supplementary Note 2. Breakpoints identified for the *plasmepsin 2-3* amplification.

The list below reports the breakpoints homology regions identified in this dataset.

PfPlasmepsin_1

9.2kbp, found in 193 samples
5' - Pf3D7_14_v3:289611-289621
3' - Pf3D7_14_v3:298782-298792

PfPlasmepsin_2

17.4kbp, found in 4 samples
5' - Pf3D7_14_v3:283034-283069
3' - Pf3D7_14_v3:300493-300522

PfPlasmepsin_3

79.9kbp, found in 2 samples
5' - Pf3D7_14_v3:283034-283069
3' - Pf3D7_14_v3:362990-363020

Supplementary Note 3. Alleles that characterize the KEL1 haplogroup.

We found the KEL1 haplotype to be characterized by specific alleles at a small number of sites in the regions flanking the *pfkelch13* gene. From an empirical analysis of our dataset, we found that a simple scoring scheme based on the genotypes at four of these sites correctly labels 250 out of 255 (98%) of samples that were identified as KEL1 by chromosome painting analyses.

This table shows five SNPs where KEL1 parasites carry a non-reference allele (different from the 3D7 reference genome), and are characteristic of that haplogroup. For each SNP, the table below reports: the flank of *pfkelch13* where the SNP is located; the position of the SNP in the 3D7 V3 chromosome 13 reference sequence; the 3D7 reference allele; the KEL1 characteristic allele; the SNP's distance from the C580Y mutation site.

Flank	Chromosome 13 Position	Allele		Distance	Notes
		Reference	KEL1		
**Left (5')**	1700345	T	C	-24,914	Optional (see below)
**Left (5')**	1717359	T	G	-7,900	
**Left (5')**	1718288	A	T	-6,971	
**C580Y**	**1725259**	**C**	**T**	-	
**Right (3')**	1739315	A	G	14,056	
**Right (3')**	1862741	G	C	137,482	

To test how informative these sites are, we scored each 580Y sample as follows. On each flank, we scanned away from the *pfkelch13* gene, counting the number of consecutive SNPs that carry the KEL1 allele (alone or heterozygous) but ignoring sites with missing genotypes; the scores of the two flanks were finally added together. When testing just two flanking SNPs on each side, scores > 2 correctly identified 98% of all KEL1 samples. Using all five flanking SNPs (i.e. including the optional SNP Pf3D7_13_v3:1700345) produces the same result using the same scoring scheme, but may be suitable when genotype missingness is high on the left flank.
